# Effect of Central Sensitization in Patients with Familial Mediterranean Fever, Axial Spondyloarthritis, and Both Diseases

**DOI:** 10.7759/cureus.45459

**Published:** 2023-09-18

**Authors:** Mehmet Nur Kaya, Özlem Kılıç, Abdullah Doğan, Sedat Yılmaz, Duygu Tecer

**Affiliations:** 1 Rheumatology, Gülhane Training and Research Hospital, Ankara, TUR

**Keywords:** fibromyalgia, central sensitization inventory, central sensitization, axial spondyloarthritis, familial mediterranean fever

## Abstract

Objective: Our aim in this study was to evaluate the level of central sensitization (CS) in patients having familial Mediterranean fever (FMF), axial spondyloarthritis (axSpA), and both diseases (axSpA/FMF).

Methods: This study included 30 FMF, 30 axSpA, 30 axSpA/FMF patients, and 30 healthy controls (HCs). The presence of CS was investigated by the Central Sensitization Inventory (CSI) questionnaire. In order to evaluate the effect of CS on patient groups, clinical features, disease activity, quality of life, sleep quality, depression, and anxiety frequency were examined. The patients were divided into groups according to the presence and severity of CS and their results were compared.

Results: The mean age of all participants was 28.4±5.7 years and 67 (55.8%) of them were male. The erythrocyte sedimentation rate (ESR) value was significantly higher in axSpA and axSpA/FMF groups than in FMF and HCs groups (p<0.001). ESR value was significantly higher in the FMF group than in the HCs group (p<0.001). C-reactive protein (CRP) value was significantly higher in the axSpA/FMF group than in the axSpA and HCs groups (p=0.001). CSI-Part A value was significantly higher in the axSpA, FMF, and axSpA/FMF group than in the HCs group (p<0.001). CSI-Part A value did not differ significantly between axSpA/FMF, axSpA, and FMF groups (p>0.05). The presence of chronic fatigue syndrome was found to be significantly higher in the FMF group than in the axSpA and HCs groups (p<0.05). Fibromyalgia syndrome was significantly higher in the axSpA/FMF group than in the axSpA, FMF, and HCs groups (p<0.05).

Conclusions: In this study, the CS score was found to be significantly higher in axSpA and FMF patients compared to the HCs group. There was no difference between the disease groups in terms of CS score.

## Introduction

Inflammatory low back pain and morning stiffness are the main symptoms of axial spondyloarthritis (axSpA), which affects the spine and mainly the sacroiliac joints. The objectives of treatment for axSpA encompass the mitigation of symptoms, including pain and stiffness, the preservation of proper posture, the prevention of spinal fusion, and the enhancement of overall quality of life. The Bath Ankylosing Spondylitis Disease Activity Index (BASDAI) or Ankylosing Spondylitis Disease Activity Score (ASDAS) are generally used for disease activity assessment in daily practice [1]. While nonsteroidal anti-inflammatory drugs (NSAIDs) are primarily recommended for the pharmacological treatment of most patients with axSpA, biologic agents are the next step in the treatment of unresponsive patients [1]. Familial Mediterranean fever (FMF) is a hereditary autoinflammatory disorder that follows an autosomal recessive pattern of inheritance. It is distinguished by recurring episodes of fever and inflammation affecting the serous membranes, which are often observed alongside the fever. Although it can occur among older individuals, patients mostly experience their first attacks in early childhood [2]. The utilization of a particular test for diagnostic purposes is not employed; rather, clinical assessment is relied upon for the diagnosis of the condition. The observed elevation of acute phase reactants during the attack, followed by their return to normal levels during the non-attack period, provides supportive evidence for the diagnosis. The primary objective of FMF treatment is to proactively avert the occurrence of attacks, mitigate the extent of inflammation, and forestall the development of amyloidosis. The initial treatment for FMF is colchicine; however, agents developed against IL-1 are used in cases of unresponsiveness to treatment [2]. Allodynia and hyperalgesia are characteristic features of central sensitization (CS), as the unpleasant pain persists after the stimulus has disappeared. Even after minor trauma, the mediators released due to inflammation activate the nociceptors in peripheral tissues and the formation of neuronal hyperexcitability as a result of increasing electrochemical discharges forms the basis of the physiology of CS [3].

The concept of CS has been put out as a physiological occurrence inside the central nervous system that leads to an increased sensitivity to both painful and painless stimuli. This heightened sensitivity is believed to arise from dysregulation and hyperexcitability of neurons. CS is a medical disorder that is distinguished by an atypical escalation in pain resulting from neuronal hyperreactivity and the impairment of both the ascending and descending pathways within the central nervous system [4]. CS affects the patient in various ways, especially the pain symptom. The primary objective of the present study was to examine the correlation between pain catastrophizing, pain severity, and patient-reported disease activity within a cohort of individuals diagnosed with rheumatoid arthritis. Notably, no significant elevation in acute phase reactants was seen, and joint ultrasonography failed to reveal any indications of joint inflammation [5]. In a separate investigation, findings indicated that 40% of individuals diagnosed with ankylosing spondylitis (AS) and subjected to a seven-year course of etanercept therapy experienced enduring discomfort, as evidenced by a pain score exceeding 4 on a scale ranging from 0 to 10 [6]. The study by Spoorenberg et al. showed that patients with AS consider their own symptoms when evaluating disease activity, but physicians also include laboratory parameters, physical examination, imaging methods, and patient's views when evaluating disease activity [7]. Concurrent pain sensitivity affects pain-related parameters in patient-based activity scores and impairs health-related measures [8]. Insufficient research has been conducted to adequately assess the impact of CS on disease activity among persons diagnosed with axSpA and FMF [8,9].

The aim of this study was to evaluate the extent of CS and its association with clinical and functional features in persons diagnosed with FMF, axSpA, and both axSpA and FMF.

## Materials and methods

Study design and sampling

The present study is characterized as a cross-sectional, observational investigation conducted at a single center. Between October 2022 and March 2023, patients followed in Gülhane Training and Research Hospital rheumatology outpatient clinics were included in the study. All individuals included in the study are above the age of 18, and the study population comprises patients diagnosed with FMF, axSpA, or both axSpA and FMF, as well as a control group of individuals without these conditions, referred to as healthy controls (HCs). The sample size for each group was 30, resulting in a total of 120 individuals. The classification criteria for axSpA as established by the ASAS were employed [10]. The patients' FMF diagnosis was established based on the Tel-Hashomer criteria [11]. Individuals between the ages of 18 and 75 who have been diagnosed with the condition of FMF and axSpA were included in the study, while those with psychiatric disorders, chronic diseases, those receiving medical treatment (e.g., duloxetine, antidepressants, antipsychotics), and other systemic inflammatory rheumatic diseases were excluded from the study. The present study received ethical approval from the Ethics Committee of Health Sciences University, Gülhane Training and Research Hospital, in accordance with the principles outlined in the Declaration of Helsinki (Date: 24.11.2022 and Decision number: 2022/159).

Clinical variables 

Information pertaining to the demographic and clinical profiles of all individuals enrolled in the study was collected, together with evaluations linked to the specific ailment under investigation. These assessments encompassed various factors such as age, gender, education level (categorized according to the cutoff value of International Standard Classification of Education level >4), current smoking status, and body mass index. The study involved the collection of data from individuals diagnosed with axSpA in relation to their HLA-B27 status, past occurrences of extra-articular symptoms, the existence of peripheral arthritis, and utilization of NSAIDs and biologic therapy. Several disease activity assessment tools, including C-reactive protein (CRP), erythrocyte sedimentation rate (ESR), ASDAS-CRP, ASDAS-ESR, and BASDAI were employed in this study. Data about Mediterranean fever (MEFV) genotyping, frequency of attacks within the preceding 3 and 6 months, specific types of the attacks, presence of amyloidosis, dosage of colchicine administered, resistance to colchicine treatment, medications employed, and levels of acute phase reactants during inter-attack periods were collected from individuals diagnosed with FMF. The International Severity Score for FMF (ISSF) activity scoring system was employed as a measure of illness activity [12].

Outcome measures 

The Central Sensitization Inventory (CSI) is comprised of two components, namely, Part A and Part B. Part A consists of a set of 25 items that are evaluated using a 5-point Likert scale. These items aim to assess the presence and severity of symptoms related to CS. Part A is classified based on the numerical range of 0-100, with consideration of the presence of symptoms related to CS. The categorization of severity levels in the context of CS is as follows: subclinical (0-29), mild (30-39), moderate (40-49), severe (50-59), and excessive (60-100) [13]. Part B investigates the presence of 10 central sensitivity syndromes [3]. The CSI questionnaire utilized in our study was the Turkish version, which had been validated by Düzce et al. [14]

The Pittsburgh Sleep Quality Index (PSQI) is a comprehensive 24-item scale utilized to assess sleep quality and disturbances experienced within the previous month. The scored 18 questions of the scale consist of 7 components. Each individual piece is evaluated using a scale that spans from 0 to 3 points. The summation of the scores from the seven individual components yields the scale score. The overall score spans from 0 to 21. A cumulative score beyond 5 signifies a substandard level of sleep quality [15]. In this study, the Turkish version of the PSQI questionnaire was employed [16]. 

The Nottingham Health Profile (NHP) is a comprehensive health assessment tool that encompasses six distinct parameters: energy level, emotional reactions, physical activity, pain, sleep, and social isolation. It comprises a total of 38 items, which collectively contribute to a comprehensive evaluation of an individual's overall health status. The overall score ranges from 0 to 600, and there exists an inverse relationship between the score achieved and the perception of a great quality of life in relation to health [17]. Küçükdeveci et al. conducted an assessment of the reliability and validity of the NHP in the Turkish population [18]. 

The Hamilton Depression Scale (HAM-D) is a standardized assessment tool comprising 17 items that inquire about the manifestations of depression encountered throughout the preceding week. The scale items pertaining to the challenges associated with initiating sleep, experiencing nocturnal awakenings, waking up prematurely in the morning, somatic symptoms, genital symptoms, weight loss, and insight were assessed on a scale ranging from 0 to 2, while the remaining items were graded on a scale ranging from 0 to 4. The maximum score attainable is 53 points. A score between 0 and 7 means you don't have depression, while a score between 8 and 15 means you have mild depression. A score between the ranges of 16 to 28 is indicative of moderate depression, whereas a score of 29 or more is indicative of severe depression [19]. HAM-D has undergone translation and validation in the Turkish language by Akdemir et al. [20].

Hamilton Anxiety Rating Scale (HAM-A) scale was developed to determine the level of anxiety in individuals and to measure the development of violence. The inventory comprises a total of fourteen items that assess a range of mental and physical symptoms. Each individual item is assigned a numerical value ranging from 0 to 4, and the cumulative score is obtained by summing the scores of all the items. When anxiety was graded according to HAM-A levels, 6-14 was considered minor, and 15 and above was considered major anxiety [21]. In this study, we used the Turkish reliability and validity of the HAM-A questionnaire [22]. 

The ISSF activity score is used to evaluate the disease activity of FMF patients. This scoring system consists of 10 items in total. Article 4 is divided into 4a and 4b. According to the definition of the 4a/4b criteria, up to 2 points, the presence of other items equals 1 point. The total score was defined as ≤2 points mild disease, 3-5 moderate disease, ≥6 severe disease. In this study, we used the validated Turkish version of the ISSF activity score [12].

Statistical analysis

The categorical variables were presented in terms of the number of cases represented as a percentage. Normally distributed variables were described using the mean and standard deviation (SD), whereas non-normally distributed data were summarized using the median. The Shapiro-Wilk test was utilized to assess the distribution of variables. The Kruskal-Wallis test was employed to analyze independent quantitative data. The Chi-Square test was utilized to analyze qualitative independent data, while the Fischer-Exact test was utilized in situations where the conditions for conducting the Chi-Square test could not be met. The statistical analyses were conducted using the statistical tool for the social sciences (SPSS) software tool, specifically version 28 (IBM Corp., Armonk, NY). Statistical significance was determined by accepting p-values that were less than 0.05.

## Results

The average age of all participants was 28.4±5.7 years, with 67 (55.8%) of them being male. Demographic, clinical, laboratory, and medications used by axSpA, FMF, and axSpA/FMF patients included in the study are shown in Table 1. ESR value was significantly higher in axSpA and axSpA/FMF groups than in FMF and HCs groups. ESR was found to be considerably elevated in the FMF group compared to the HCs group (p<0.001). There was no observed statistically significant difference observed among the remaining groups (p>0.05). CRP value was significantly higher in the axSpA/FMF group than in the axSpA and HCs groups. CRP value in the FMF group was significantly higher than the HCs group (p=0.001). No statistically significant differences were seen among the other groups (p>0.05) (Table 1). 

**Table 1:** Patient characteristics of the study population of the axSpA, FMF, axSpA/FMF, and HCs groups (n=120) Values are presented in n (%), mean ± (SD), or median (IQR); a: Defined as International Standard Classification of Education (ISCED) level >4; b: Defined as a swollen joint count of ≥1; c: Defined as Maastricht Ankylosing Spondylitis Enthesitis Score (MASES)≥1; d: Biologicals include TNF-A inhibitors and the IL-17A inhibitor secukinumab: IBD: Inflammatory Bowel Disease; NSAID: Nonsteroidal Anti-Inflammatory Drugs; BMI: Body Mass Index; ESR: Erythrocyte Sedimentation Rate; CRP: C-Reactive Protein; ASDAS-CRP: Ankylosing Spondylitis Disease Activity Score with CRP; ASDAS-ESR: Ankylosing Spondylitis Disease Activity Score with ESR; BASDAI: Bath Ankylosing Spondylitis Disease Activity Index; axSpA: Axial Spondyloarthritis; FMF: Familial Mediterranean Fever; HCs: Healthy Controls; ISSF: International Severity Score for FMF

Characteristics		axSpA	FMF	axSpA/FMF	HCs	p
Age, years	mean ± SD	27.9 ± 5.6	27.3 ± 3.6	29.1 ± 5.9	29.3 ± 7.2	0.638
	median (IQR)	27.5 (7.3)	28 (4.5)	29 (13)	27 (10.3)	-
Male	n (%)	15 (50)	16 (53.3)	16 (53)	20 (66.7)	0.574
High education level^a^	n (%)	24 (80)	23 (76.7)	18 (60)	10 (33.3)	0.071
BMI, kg/m^2^	mean ± SD	24.2 ± 4.1	26.1 ± 2.7	25.1 ± 2.7	25.2 ± 3.7	0.397
	median (IQR)	24.2 (6.3)	26.5 (4.9)	25.4 (3.6)	26.7 (4.6)	-
Current smoker	n (%)	13 (43.3)	11 (36.7)	14 (46.8)	21 (70)	0.426
Time since diagnosis, years	mean ± SD	4.9 ± 2.1	6.9 ± 5.6	6 ± 2.6	-	-
	median (IQR)	5 (3)	4 (10)	5 (5)	-	-
Age at diagnosis, years	mean ± SD	22.9 ± 4.8	20.1 ± 6.8	23 ± 5.1	-	-
	median (IQR)	22.5 (6)	18 (11.8)	21 (11)	-	-
HLA-B27	n (%)	13 (43.3)	-	12 (40)	-	-
History of IBD	n (%)	4 (13.3)	-	3 (10)	-	-
History of uveitis	n (%)	2 (6.6)	-	1 (3.3)	-	-
History of psoriasis	n (%)	6 (20)	-	9 (30)	-	-
Current peripheral arthritis^b^	n (%)	13 (43.3)	-	10 (33.3)	-	-
Current entheseal involvement^c^	n (%)	22 (73.3)	-	8 (26.6)	-	-
NSAID	n (%)	29 (96.7)	-	27 (90)	-	-
Sulfasalazin	n (%)	7 (23.3)	-	10 (33.3)	-	-
Biological^d^	n (%)	15 (50)	-	9 (30.3)	-	-
ASDAS-CRP	mean ± SD	1.9 ± 0.5	-	2.1 ± 0.5	-	-
	median (IQR)	1.9 (0.7)	-	2.1 (0.7)	-	-
ASDAS-ESR	mean ± SD	2.3 ± 0.5	-	2.4 ± 0.6	-	-
	median (IQR)	2.2 (0.8)	-	2.5 (0.8)	-	-
BASDAI	mean ± SD	3.4 ± 1.3	-	3.7 (1.4)	-	-
	median (IQR)	3.3 (1.9)	-	3.6 (1.6)	-	-
Levels between attacks	-	-	-	-	-	-
ESR (mm/h)	mean ± SD	17.6 ± 8.5	12.9 ± 7.4	19 ± 7.1	8.5 ± 3.8	<0.001
	median (IQR)	15.5 (10)	12 (7)	19.7 (9.1)	8 (4)	-
CRP (mg/L)	mean ± SD	7.4 ± 7.9	10.3 ± 10.1	15.3 ± 14.5	4 ± 2.1	0.001
	median (IQR)	4 (5)	6 (9)	9 (17)	4.5 (1.9)	-
Attack features	-	-	-	-	-	-
Fever (≥38°C)	n (%)	-	14 (46.7)	14 (46.7)	-	-
Abdominal pain	n (%)	-	28 (93.3)	24 (80)	-	-
Chest pain	n (%)	-	13 (43.3)	7 (23.3)	-	-
Arthritis	n (%)	-	2 (6.7)	10 (33.3)	-	-
Myalgia	n (%)	-	4 (13.3)	6 (20)	-	-
Erythema	n (%)	-	4 (13.3)	6 (20)	-	-
Colchicine dose (mg/day)	mean ± SD	-	1.5 ± 0.5	1.5 ± 0.5	-	-
	median (IQR)	-	-	-	-	-
Colchicine resistance	n (%)	-	6 (20)	7 (23.3)	-	-
Anakinra	n (%)	-	1 (3.3)	4 (13.3)	-	-
Canakinumab	n (%)	-	1 (3.3)	0 (0)	-	-
Number of attacks	-	-	-	-	-	-
In the last 3 months	mean ± SD	-	0.4 ± 0.8	1.0 ± 0.8	-	-
	median (IQR)	-	0 (0)	1 (2)	-	-
In the last 6 months	mean ± SD	-	1.2 ± 1.5	1.5 ± 1.4	-	-
	median (IQR)	-	1 (1)	1 (1)	-	-
In the last 12 months	mean ± SD	-	2 ± 2.1	2.6 ± 2.2	-	-
	median (IQR)	-	1 (1.2)	2 (2)	-	-
ISSF	mean ± SD	-	3 ± 2.4	4.0 ± 2.1	-	-
	median (IQR)	-	3 (5)	4 (3)	-	-

The CSI-Part A value demonstrated a statistically significant elevation in the groups with axSpA, FMF, and axSpA/FMF in comparison to the group of HCs (p<0.001). No statistically significant difference was seen in the CSI-Part A value among the patient groups with axSpA/FMF, axSpA, and FMF (p>0.05). NHP value was significantly higher in the axSpA/FMF, axSpA, and FMF group than in the HCs group (p<0.001). There was no statistically significant difference observed among the remaining groups (p>0.05). PSQI scale was found to be significantly higher in the axSpA/FMF and axSpA groups than in the HCs group (p<0.001). PSQI scale did not differ significantly between axSpA/FMF, axSpA, and FMF groups (p>0.05). HAM-A scale was found to be significantly higher in the axSpA, FMF, and axSpA/FMF group than in the HCs group (p<0.001). There was not a statistically significant distinction observed in the HAM-A scale scores when comparing the axSpA/FMF, axSpA, and FMF groups (p>0.05). The HAM-D scale was significantly higher in the axSpA/FMF, axSpA, and FMF group than in the HCs group. The HAM-D scale was found to be significantly higher in the axSpA/FMF group than in the axSpA and FMF groups (p<0.001). There was no statistically significant difference observed in the HAM-D scale scores between the groups with axSpA and FMF (p>0.05) (Table 2). 

**Table 2:** Comparison of Central Sensitization Inventory, Pittsburgh Sleep Quality Index, Nottingham Health Profile, Hamilton Depression Scale, and Hamilton Anxiety Rating Scale scores between patients with axSpA, FMF, axSpA/FMF, and HCs groups axSpA: Axial Spondyloarthritis; FMF: Familial Mediterranean Fever; HCs: Healthy Controls; CSI: Central Sensitization Inventory; NHP: Nottingham Health Profile; PSQI: Pittsburgh Sleep Quality Index; HAM-A: Hamilton Anxiety Rating Scale; HAM-D: Hamilton Depression Scale

		axSpA	FMF	axSpA/FMF	HCs	p
CSI Part A	mean ± SD	41.0 ± 19.8	41.5 ± 22.0	50.1 ± 19.6	15.0 ± 9.4	<0.001
	median (IQR)	35.0 (32)	40.0 (36.7)	47.0 (31)	12.0 (1)	-
NHP	mean ± SD	174.4 ± 104.3	168.2 ± 137.4	164.5 ± 74.7	57.7 ± 36.8	<0.001
	median (IQR)	129.5 (172)	85.9 (251.6)	171 (80.8)	47.0 (6.6)	-
PSQI	mean ± SD	9.5 ± 3.0	8.3 ± 4.1	10.0 ± 3.5	6.3 ± 2.9	<0.001
	median (IQR)	9.0 (5)	8.0 (7)	9.0 (6)	5.0 (1.3)	-
HAM-A	mean ± SD	11.1 ± 5.3	12.8 ± 6.4	11.8 ± 5.3	6.9 ± 4.5	<0.001
	median (IQR)	11.0 (7)	10.0 (11.5)	9.5 (9)	6.0 (3.5)	-
HAM-D	mean ± SD	14.5 ± 7.9	14.8 (8.2)	18.6 ± 6.8	10.0 ± 6.8	<0.001
	median (IQR)	12.0 (16)	15.0 (17.5)	19.0 (11)	7.0 (10.3)	-

The presence of chronic fatigue syndrome was found to be significantly higher in the FMF group than in the axSpA and HCs groups (p<0.05). No statistically significant differences were observed in the prevalence of chronic fatigue syndrome among the remaining groups. The prevalence of fibromyalgia syndrome was shown to be considerably greater in the group with both axSpA and FMF compared to the groups with only axSpA, FMF, and HCs (p<0.05). The presence of fibromyalgia syndrome was significantly higher in the axSpA and FMF groups than in the HCs group (p<0.05). There was no significant difference observed in the prevalence of fibromyalgia syndrome between the groups with axSpA and FMF (p>0.05). The depression rate was significantly higher in the axSpA/FMF, axSpA, and FMF groups than in the HCs group (p<0.05). There was no observed statistically significant difference among the remaining groups (p>0.05) (Table 3).

**Table 3:** Frequency of central sensitization syndrome in axSpA, FMF, axSpA/FMF, and HCs groups axSpA: Axial Spondyloarthritis; FMF: Familial Mediterranean Fever; HCs: Healthy Controls; CSI: Central Sensitization Inventory

CSI Part B		axSpA	FMF	axSpA/FMF	HCs	p
1-Restless leg syndrome	n (%)	5 (16.7)	4 (13.3)	5 (16.7)	3 (10.0)	0.860
2-Chronic fatigue syndrome	n (%)	4 (13.3)	11 (36.7)	5 (16.7)	2 (6.7)	0.018
3-Fibromyalgia	n (%)	16 (53.3)	11 (36.7)	5 (16.7)	2 (6.7)	<0.001
4-Temporomandibular joint disorder	n (%)	0 (0.0)	0 (0.0)	0 (0.0)	0 (0.0)	-
5-Migraine or tension headaches	n (%)	4 (13.3)	2 (6.7)	5 (16.7)	4 (13.3)	0.694
6-Irritable bowel syndrome	n (%)	13 (43.7)	7 (23.3)	15 (50.0)	3 (10.0)	0.003
7-Multiple chemical sensitivities	n (%)	0 (0.0)	0 (0.0)	0 (0.0)	0 (0.0)	-
8-Neck injury (including whiplash)	n (%)	0 (0.0)	0 (0.0)	0 (0.0)	0 (0.0)	-
9-Anxiety or panic attacks	n (%)	4 (13.3)	2 (6.7)	4 (13.3)	1 (3.3)	0.440
10-Depression	n (%)	16 (53.3)	16 (53.3)	15 (50.0)	5 (16.7)	0.009

## Discussion

In the literature, it has been determined that CSI scores are correlated with high disease activities in axSpA and FMF diseases, as well as with biopsychosocial parameters [8,9]. The objective of this study was to assess the correlation between the level of CS and several clinical and functional indicators in individuals diagnosed with FMF, axSpA, and axSpA/FMF. However, we also evaluated whether the axSpA/FMF patients differed from the FMF and axSpA patient groups in terms of CS level. This is the first study in the literature among these disease groups. In this cross-sectional study, the CSI Part A score was found to be higher in the axSpA, FMF, and axSpA/FMF disease groups compared to the HCs group, but no statistically significant difference was found between the disease groups. There was no significant difference between the ASDAS-CRP, ASDAS-ESR, and BASDAI disease activity scores in the axSpA and axSpA/FMF patient groups with a CSI score >40. HAM-A, HAM-D, and NHP scores in the axSpA, FMF, and axSpA/FMF disease groups were found to be higher than the HCs group, but no significant difference was found between the disease groups. PSQI score was higher in the axSpA and axSpA/FMF disease groups than in the HCs group, however, no statistically significant difference was found between the FMF and HCs groups. In the study including patients with axSpA, it was shown that the PSQI score was comparatively higher in the patient subgroup with a CSI of 40 or above, as opposed to the patient subgroup with a CSI below 40 [23]. Moreover, previous studies have demonstrated that individuals with FMF exhibit reduced sleep quality and elevated levels of anxiety, depression, and fatigue when compared to individuals without the condition [24]. The current status of insomnia may be influenced by the presence of a chronic disease, as well as the occurrence of nocturnal pain.

CS can be clinically recognized by the presence of symptoms such as hyperesthesia and allodynia. Nevertheless, CS can present itself through a diverse range of cognitive, emotional, and physical manifestations. Therefore, it is crucial to thoroughly examine the domain of CS within its whole biopsychosocial framework, which includes both clinical implementation and scholarly exploration [8].

In the study which included 182 axSpA, ASDAS-CRP, ASDAS-ESR, and BASDAI disease activity scores were found to be high in the patient group with a CSI Part A score of >40 [8]. Karlıbel et al. conducted a study employing a control group to assess the impact of CS on axSpA [23]. The results indicated that axSpA patients with CS exhibited notably elevated BASDAI, ASDAS-CRP, and PSQI scores in comparison to axSpA patients without CS. In another study conducted in axSpA, clinical conditions such as pain, BASDAI, disease activity score, fatigue, sleep quality, depression, and functional status were correlated with the CSI score [25]. In our study, there was no significant difference between the axSpA and axSpA/FMF groups in ASDAS-CRP, ASDAS-ESR, BASDAI activity scores, anxiety, depression, sleep quality, and functional status. No significant distinction was seen in relation to CS between the aforementioned groups. CS was found to be significantly higher in the axSpA, axSpA/FMF, and FMF groups compared to the HCs group.

CS has introduced a novel viewpoint to a range of clinical factors, particularly in relation to pain. Inflammatory mediators trigger the sensitization process and play a role in all its steps, making CS more prominent in rheumatic diseases. One of the primary factors to consider is the involvement of FMF-associated pyrin and other genes, which are believed to influence sensitization mechanisms and contribute to the development of intense inflammatory burden via persistent subclinical inflammation and recurring systemic assaults [26].

The study done by Yücel et al. aimed to assess the presence of CS in a sample of 100 patients diagnosed with FMF [9]. The researchers examined many factors including disease activity, anxiety levels, depression levels, sleep quality, and functional status. The findings revealed that patients with high scores on the CSI exhibited higher levels of disease activity, anxiety, depression, sleep disturbances, and worse functional status compared to patients with low CSI scores. In our study, CS, disease activity, anxiety, depression, sleep quality, and functional status were found to be significantly higher in FMF patients compared to the HCs group.

CS syndromes encompass a collection of syndromes that lack structural abnormalities and involve the overexcitability of central neurons due to diverse synaptic and neurotransmitter activities, independent of organic pathology. These CS syndrome disorders share several common characteristics, including pain, fatigue, disrupted sleep, heightened sensitivity to both painful and non-painful stimuli, comorbidity, paresthesia, and psychosocial disturbances [27]. Fibromyalgia syndrome is a clinical manifestation with an unclear origin, presenting as persistent and widespread pain, fatigue, sleep disturbances, cognitive impairments, and somatic symptoms. During physical examination, tender points that elicit pain upon palpation are identified, while there are no specific laboratory findings associated with this condition [28]. When considering the differential diagnosis of fibromyalgia syndrome, it is important to take into account rheumatological diseases. In a study of 89 patients with CS syndrome, 47% of the patients reported tension headaches/migraines, 47% as myofascial pain syndrome and 38% as fibromyalgia [29]. The prevalence of CS syndrome in patients with Behçet's illness was seen to be 48.9% out of a sample size of 88 individuals. The observed proportion had a significantly higher magnitude when compared to the control group. In this study, the most common CS syndrome was migraine or tension headaches, however, the observed results did not demonstrate statistical significance in comparison to the control group. Restless leg syndrome and depression have been observed to have a significantly greater prevalence in individuals diagnosed with Behçet's disease when compared to a control group, indicating the presence of CS syndromes in this patient population [30]. The most common CS syndrome in FMF patients is tension headaches/migraines, and it was found to be significantly higher in the group with a high CS score [9]. In our study, fibromyalgia was found most frequently in axSpA patients, and chronic fatigue syndrome and fibromyalgia were the most common in FMF patients. In addition, irritable bowel syndrome (IBS) was found most frequently in axSpA/FMF patients and differed from other studies.

One of the strongest aspects of this study is that there is no significant difference between the CS scores in the evaluation of axSpA and FMF patients according to the disease group with both diseases. It is especially important to include the control group. The primary limitations of this study pertain to its utilization of a cross-sectional design and its inclusion of a very limited patient sample size. Furthermore, the utilization of quantitative sensory tests, pressure pain thresholds, and monofilaments can serve as effective methods for identifying patients who are very likely to have CS.

## Conclusions

The results of our study indicate that the CS score was considerably elevated in patients with axSpA and FMF compared to the control group consisting of healthy individuals. Nevertheless, the study did not see a statistically significant difference in the CS score between individuals with axSpA/FMF and those with either axSpA or FMF. Evaluation of CS scores and CS syndromes in the evaluation of these diseases is important in terms of treatment management. Conducting prospective studies with a larger number of patients will contribute more to the literature.
